# Black Esophagus and Recurrence of Duodenal Ulcers: Two Signs of the Same Pathogenic Pathway? A Case Report

**DOI:** 10.3390/reports6030037

**Published:** 2023-08-07

**Authors:** Daniele Balducci, Claudia Quatraccioni, Luigi Maria Daretti, Michele Montori, Emanuele Bendia, Luca Maroni, Antonio Benedetti

**Affiliations:** 1 Clinic of Gastroenterology, Hepatology, and Emergency Digestive Endoscopy, Università Politecnica delle Marche, 60126 Ancona, Italy; 2 SOD Malattie Apparato Digerente, Endoscopia Digestiva, Malattie Infiammatorie Croniche Intestinali, Ospedali Riuniti, 60126 Ancona, Italy

**Keywords:** black esophagus, circumferential esophageal necrosis, duodenal ulcer, upper gastrointestinal bleeding, coffee ground emesis, case report

## Abstract

Black esophagus or acute esophageal necrosis is characterized by circumferential black discoloration of the distal esophageal mucosa. It is a rare condition with a multifactorial pathogenesis, and its most common clinical presentation is acute upper gastrointestinal bleeding. It usually affects elderly patients with multiple comorbidities and is associated with a high mortality rate. This is a case report of a 90-year-old man with multiple comorbidities, including diabetes mellitus, atrial fibrillation with complete atrioventricular block, and a history of ischemic stroke, who presented to the emergency department for a syncopal episode followed by coffee ground emesis. Thoraco-abdominal computer tomography showed thickening of the distal esophagus and ruled out major complications such as perforation. The following esophagogastroduodenoscopy showed black circumferential necrosis of the mid and distal esophagus. Multiple irregular ulcers with black necrotic areas were also present in the bulb and second duodenal portion. During the hospitalization, the patient was treated with PPI, NPO nutrition, and broad-spectrum antibiotics with benefits. Two months later, the patient returned to the emergency department due to a new episode of hematemesis with endoscopic evidence of esophageal stricture without necrosis and recurrence of duodenal ulcers. After a few days, the patient died due to worsening of the underlying comorbidities. A black esophagus is associated with duodenal ulcers, which may recur and are possibly due to a common ischemic origin. In this case report, we explore the potential link between black esophagus and duodenal ulcers, discussing the underlying mechanisms and relevant literature supporting this association.

## 1. Introduction

Black esophagus, also known as acute esophageal necrosis (AEN) or acute necrotizing esophagitis, is a rare life-threatening condition described for the first time by Goldenberg et al. [1] in 1990. While the reported incidence is low, ranging from 0.01% to 0.29% [2,3], the overall fatality rate is 32% [4]. AEN is characterized by severe circumferential injury to the esophageal mucosa, with black discoloration that stops at the gastroesophageal junction [5,6]. While the pathogenesis seems multifactorial, the most common reported etiology is ischemia [6]. In fact, it is hypothesized that the organ injury can be caused by a transient or prolonged hemodynamic compromise in a susceptible individual with an underlying vascular disease and impaired mucosal defenses [6]. However, while there is not a comprehensive understanding of the underlying mechanism of action, viral infection, massive reflux of gastric secretions, hypothermia, antibiotic hypersensitivity, and corrosive trauma can cause AEN [1,7]. The most common presentation is acute upper gastrointestinal (GI) bleeding. Possible complications include distal esophagus stricture, perforation, mediastinitis, and death, with an overall mortality rate of 32% [8]. This syndrome usually appears in older patients with multiple comorbidities, such as hypertension, coronary artery disease, diabetes mellitus, and malignancy [4]. While AEN remains an unusual cause of upper GI hemorrhage, the incidence and prevalence of the condition are believed to be underestimated. AEN was identified in up to 6% of the cases admitted in hospital for coffee ground emesis, hematemesis, or melena [9].

In this paper, we describe a case of a 90-year-old adult male with multiple comorbidities, such as diabetes mellitus, atrial fibrillation with complete atrioventricular block, and a history of ischemic stroke, who developed a black esophagus along with duodenal ulcers. The presented case had a relapse after initial improvement confirmed by upper endoscopic examination. Clinical characteristics and endoscopic and histopathological findings with follow-up are presented.

## 2. Detailed Case Description

### 2.1. Chief Complaints

A 90-year-old Caucasian male presented to the emergency department of our hospital after a syncopal episode followed by coffee ground emesis.

### 2.2. History of Present Illness

The patient’s symptoms started 2 weeks prior with loss of appetite and reduction in food and fluid intake due to dysphagia. He denied the use of alcohol or non-steroidal anti-inflammatory agents and the ingestion of harmful substances (i.e., caustic ingestion).

### 2.3. History of Past Illness

The patient had a significant medical history of diabetes mellitus, ischemic stroke, atrial fibrillation with complete atrioventricular block and subsequent pacemaker implantation, intestinal resection for complicated diverticulitis, and radical prostatectomy.

### 2.4. Medical Therapy at Admission

The patient’s habitual medical therapy consisted of Aspirin 100 mg, furosemide/spironolactone 25/37 mg, and insulin.

### 2.5. Physical Examination

The patient presented with cachexia and somnolence. The abdominal examination was unremarkable, but reduced breath sounds were noted at the bases. Body temperature was 37.5 °C, heart rate was 90 beats per min, respiratory rate was 17 breaths per minute, blood pressure was 122/74 mmHg, and oxygen saturation in room air was 96%.

### 2.6. Laboratory Examinations

Blood analysis showed leukocytosis, mild anemia with reduced hematocrit, and normal platelet count. Prothrombin activity was slightly below normal, whereas fibrinogen was elevated. Serum C-reactive protein and serum creatinine levels were mildly increased. Hyperglycemia and hypoalbuminemia were also present. Laboratory examinations results are covered in detail in Table 1.

### 2.7. Imaging Examination

**Figure 1:**
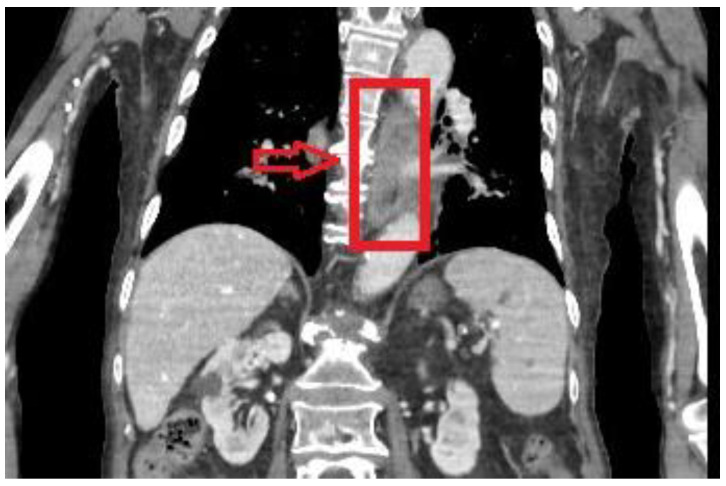
Computed tomography showing perieto-mucosal thickening of the medium–distal tract of the esophagus.

While in the emergency department, a thoraco-abdominal computer tomography (CT) scan was performed and revealed diffuse parieto-mucosal thickening of the medium-distal tract of the esophagus with edematous imbibition of the mediastinal adipose tissue (Figure 1). Celiac tripod as well as superior mesenteric artery were patent. Due to imaging findings and clinical presentation, neoplastic degeneration or an inflammatory process were mainly included in the differential diagnosis.

### 2.8. Further Diagnostic Work-Up

**Figure 2:**
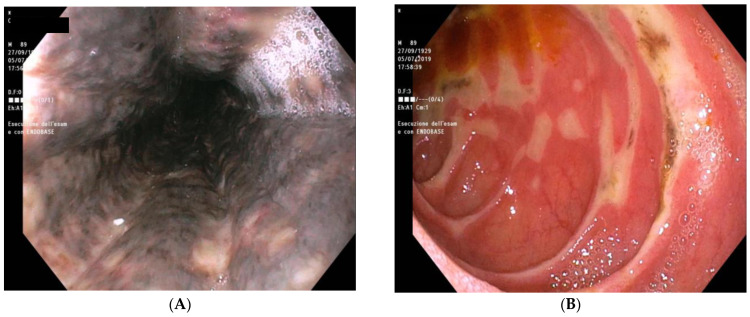
Esophagogastroduodenoscopy performed at first admission to the emergency department. (**A**) Black circumferential necrosis of the mid and distal esophagus; (**B**) ulcers with black necrotic areas in the bulb and second duodenal portion.

Further evaluation included an esophagogastroduodenoscopy (EGD) that demonstrated black circumferential necrosis of the mid and distal esophagus, which abruptly terminated at the gastroesophageal junction (Figure 2A). Multiple irregular ulcers with black necrotic areas were also present in the bulb and second duodenal portion (Figure 2B). Esophageal biopsies showed necrotic debris without fungal hyphae or other pathogens.

### 2.9. Final Diagnosis

Due to clinical presentation, typical endoscopic findings, and histopathological analyses, the final diagnosis of the presented case was AEN, also known as “black esophagus”.

### 2.10. Treatment

The patient was managed conservatively with intravenous fluid, a proton pump inhibitor (PPI), and broad-spectrum antibiotics. In particular, the patient was started on pantoprazole 40 mg IV twice daily, sucralfate 1 g every 6 h, and piperacillin/tazobactam 4.5 g IV adjusted according to creatinine clearance. At first, the diet was managed with nil-per-os (NPO) total parenteral nutrition (TPN) and advanced accordingly based on patient tolerability. Upon completion of 10 days of therapy, a subsequent EGD showed marked improvement of the esophageal necrosis and complete healing of the duodenal ulcers. The patient was, therefore, discharged on oral pantoprazole and a soft diet. Blood analysis at discharge showed normalization of white cell count and serum C-reactive protein.

### 2.11. Outcome and Follow-Up

**Figure 3:**
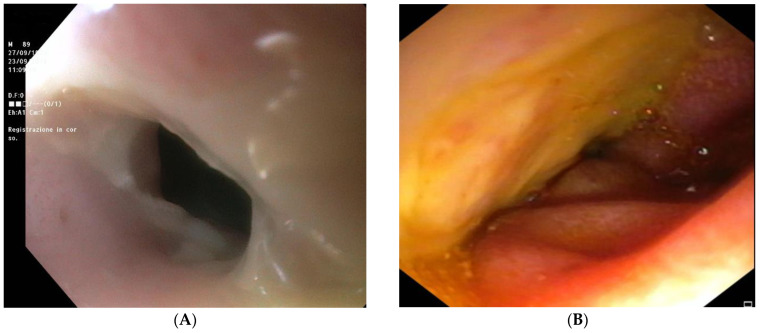
Esophagogastroduodenoscopy performed at second admission to the emergency department. (**A**) Distal esophageal stenosis without necrosis; (**B**) extensive ulcerations of duodenum.

The patient was readmitted 2 months later for recurrence of coffee ground emesis. EGD revealed the presence of a distal esophageal stenosis with no signs of necrosis (Figure 3A); however, exploration of the duodenum with a 5.9 mm gastroscope showed extensive ulcerations without active bleeding (Figure 3B). The patient died a few days later due to worsening of the general condition.

## 3. Discussion

AEN is a rare condition with unknown true prevalence. Retrospective endoscopy analysis in patients who underwent upper GI endoscopy for any reason reported an incidence of 0.01–0.29% [2,3], whereas an interesting study from Japan reported AEN as the fourth cause of upper GI bleeding with a prevalence of 6% [9]. Risk factors for the development of this syndrome are male sex, old age, alcohol abuse, non-steroidal anti-inflammatory drug use, diabetes mellitus, ketoacidosis, renal insufficiency, sepsis, cardiopulmonary disease, and cancer [4,10,11,12,13,14,15].

The exact etiology of this disorder is largely unknown and likely multifactorial. Nonetheless, hypoperfusion, weakened local defense barrier function, and massive reflux of gastric content seem to play a role [4]. AEN has also been associated with thromboembolic events in patients with underlying prothrombotic conditions such as cancer, anticardiolipin antibody syndrome, and vascular disease [9,16,17,18]. Duodenal involvement with ulcers is not uncommon, possibly due to a common blood supply from the celiac axis [19,20,21].

The typical presentation is upper GI bleeding with coffee ground emesis, melena, or hematemesis, which account for 90% of cases; however, AEN may also be asymptomatic. It can be associated with other symptoms such as dysphagia, nausea, vomiting, abdominal pain, fever, and syncope [4,8,22]. Laboratory findings may reveal an increase in white cell count, anemia, elevated CRP, elevated creatinine, hypoalbuminemia, and hyperglycemia [6,23,24]. CT scans can show a thickened distal part of the esophagus, sometimes associated with hiatal hernia and stomach distension. CT scan is also important to rule out serious complications, such as perforation, in rapidly decompensating patients [25].

Endoscopic findings are usually sufficient to make a diagnosis since the predilection of the distal esophagus is a typical feature of this condition [4], even if proximal involvement as also been reported [26,27]. EGD usually shows a circumferential, diffused black mucosa of the esophagus, which abruptly stops at the Z-line. EGD may also document signs of active bleeding, “coffee grounds” material in the stomach, duodenal ulcers, erosions, and edema [4,19,21]. Usually, esophageal biopsies are not necessary for making a proper diagnosis but can be supportive in ruling out other etiologies like bacterial, fungal, and viral infections [28]. Histopathological analyses typically show necrotic debris along with mucosal and submucosal necrosis [22].

The progression of esophageal injury has been classified in three progressive stages. Stage 0 is characterized by no signs of necrosis. Stage 1 is described as acutely damaged esophagus with the peculiar black mucosa’s endoscopic appearance. The healing phase starts with stage 2 and can be identified as residual black areas with exudates and necrotic debris upon pink mucosa. Normal endoscopic appearance (stage 3) usually occurs after 1–2 weeks [4].

Although there are no clear guidelines, the management is usually conservative and consists of the treatment of underlying diseases, intravenous hydration, PPIs, and TPN in case of poor oral tolerance [8,29]. Even if the empirical use of broad-spectrum antibiotics is widely adopted, some reports have shown a potential link between the development of AEN and their use [30]. Proper nutrition is critical for the successful recovery of the esophageal mucosa. As soon as the condition is diagnosed, the patient should undergo TPN. Enteral feeding is not recommended due to the risk of perforation. In many cases, oral nutrition can be resumed in a few days and can advance depending on the patient’s tolerance [31]. Surgical intervention is usually limited but mandatory in case of a perforated esophagus [25].

Complications can vary from stricture, stenosis, and abscesses to life-threatening conditions such as tracheoesophageal fistula and perforation [1]. Perforation incidence is reported as high as 6.8% and should be considered in patients with rapidly deteriorating clinical conditions, as prompt therapy (intravenous antibiotics and surgical intervention) can be life-saving [4]. Stenotic areas or strictures are the consequences of the reparative process of AEN and can be seen in more than 10% of patients. Medical therapy with PPI and endoscopic dilatation, when needed, usually relieve symptoms.

While the overall mortality rate is 32% [4], prognosis mostly depends on age, underlying condition, and baseline health status of the patient. In fact, AEN-specific mortality is significantly lower (<6%) [4]. On the other hand, relapse of AEN after recovery is seldom described [6].

Our patient presented to the emergency department with typical symptoms such as a syncopal episode followed by coffee ground emesis. The patient had also a history of loss of appetite and dysphagia for two weeks prior to the presentation. While our patient denied the use of alcohol, non-steroidal anti-inflammatory agents, or ingestion of harmful substances, he had multiple risks factor consistent with published literature such as male sex, old age, diabetes mellitus, and cardiovascular disease.

Based on the clinical presentation, endoscopic findings, and histopathological analyses, the final diagnosis was established as AEN, commonly known as “black esophagus”. As in other cases published in the literature [6], the patient had a marked hypoalbuminemia, which reflected a poor nutritional status. The patient also presented hyperglycemia, which may suggest a not properly managed diabetes, but it may also occur independently due to the systemic inflammatory response associated with AEN [6]. Malnourishment and microvascular insufficiency due to diabetes could lead to weakened barrier defense mechanisms and mucosal damage [6].

The presented case highlights the challenges and management of AEN. The initial conservative treatment approach showed promising results, with significant improvement observed in the esophageal necrosis. However, the recurrence of symptoms and the presence of esophageal stenosis and extensive duodenal ulcerations during the follow-up indicated a more complicated course of the disease. The outcome of the case underscores the severity of AEN and the potential for adverse outcomes, especially in elderly patients with significant comorbidities.

Given the past medical history of ischemic stroke, diabetes mellitus, and atrial fibrillation, the patient was at high risk of thromboembolic events and weakened mucosal defenses mechanisms, which are involved in the pathogenesis of this condition, as previously discussed. Gastroduodenal ischemia is commonly linked to vascular processes that alter microcirculation, leading to hypoxia and reperfusion injury, such as chronic splanchnic syndrome, hemodynamic shock, thromboembolism, and iatrogenic complications, among other causes [32]. Even if we cannot define a causal relationship, we speculate that the recurrence of duodenal ulcers was due to the relapse of an ischemic injury. This reinforces the notion that the treatment of the underlying diseases, whenever possible, is the cornerstone of proper management.

Further research and investigations are needed to better understand the pathophysiology, risk factors, and optimal management strategies for AEN, especially in cases with recurrent or refractory disease. Additionally, exploring potential interventions to prevent or mitigate complications such as esophageal stenosis and extensive ulcerations could improve patient outcomes in the future.

## 4. Conclusions

Even if esophagus stricture is a well-known complication of AEN, to our knowledge, duodenal ulcer recurrence in AEN has never been reported. We speculate that prothrombotic conditions, such as atrial fibrillation, vasculopathy, and diabetes mellitus, may play a major role in this recurrence, but further studies are needed in order to properly verifying this association. Proper management of these underlying conditions is paramount to improve outcomes and lower recurrence rate in patient with AEN.

## Figures and Tables

**Table 1:** Laboratory examinations.

Laboratory Test	Value	Normal Range
White Blood Cells	24 × 10^3^/mmc	4–10 × 10^3^/mmc
Hemoglobin	11.8 g/dL	12.5–17 g/dL
Hematocrit	35.5%	40–50%
Platelets	204 × 10^3^/mmc	150–400 × 10^3^/mmc
Prothrombin activity	62%	70–130%
Fibrinogen	647 mg/dL	200–450 mg/dL
C-reactive Protein	2.1 mg/dL	Up to 0.6 mg/dL
Creatinine	1.61 mg/dL	0.6–1.4 mg/dL
Fasting glucose	334 mg/dL	60–110 mg/dL
Albumin	2.39 g/dL	4.02–4.76 g/dL

## Data Availability

The data presented in this study are available on request from the corresponding author. The data are not publicly available due to privacy.
